# Antitumor efficacy of combined CTLA4/PD-1 blockade without intestinal inflammation is achieved by elimination of FcγR interactions

**DOI:** 10.1136/jitc-2020-001584

**Published:** 2020-10-30

**Authors:** David Bauché, Smita Mauze, Christina Kochel, Jeff Grein, Anandi Sawant, Yulia Zybina, Wendy Blumenschein, Peng Yang, Lakshmanan Annamalai, Jennifer H Yearley, Juha Punnonen, Edward P Bowman, Alissa Chackerian, Drake Laface

**Affiliations:** 1 Discovery Oncology, Merck & Co. Inc, South San Francisco, California, USA; 2 Molecular Discovery, Merck & Co. Inc, South San Francisco, California, USA; 3 Anatomic Pathology, Merck & Co. Inc, South San Francisco, California, USA

**Keywords:** immunotherapy, inflammation, CTLA-4 antigen

## Abstract

**Background:**

Programmed cell death protein 1 (PD-1) and CTLA4 combination blockade enhances clinical efficacy in melanoma compared with targeting either checkpoint alone; however, clinical response improvement is coupled with increased risk of developing immune-related adverse events (irAE). Delineating the mechanisms of checkpoint blockade-mediated irAE has been hampered by the lack of animal models that replicate these clinical events.

**Methods:**

We have developed a mouse model of checkpoint blockade-mediated enterocolitis via prolonged administration of an Fc-competent anti-CTLA4 antibody.

**Results:**

Sustained treatment with Fc-effector, but not Fc-mutant or Fc-null, anti-CTLA4 antagonist for 7 weeks resulted in enterocolitis. Moreover, combining Fc-null or Fc-mutant CTLA4 antagonists with PD-1 blockade results in potent antitumor combination efficacy indicating that Fc-effector function is not required for combination benefit.

**Conclusion:**

These data suggest that using CTLA4 antagonists with no Fc-effector function can mitigate gut inflammation associated with anti-CTLA4 antibody therapy yet retain potent antitumor activity in combination with PD-1 blockade.

## Introduction

Immunotherapy has taken a prominent role in the treatment of a number of cancer indications.^1 2^ Recent clinical successes using antibody blockade of immune checkpoint inhibitory receptors expressed on T cells such as CTLA4 or PD-(L)1 have transformed the treatment options for some patients with cancer. As monotherapies, these antibodies have demonstrated enhanced antitumor responses and beneficial clinical outcomes in controlled randomized clinical trials.^3 4^ Additional efficacy is achieved with combination approaches.^4–6^ Multiple combinations are currently being investigated in patients and several have been approved, including the combination of anti-PD-1 and anti-CTLA4 in metastatic melanoma, renal cell carcinoma (with platinum-doublet chemotherapy), and non-small cell lung cancer (www.fda.gov).

The incidence of serious immune-related adverse events (irAE) associated with immune checkpoint blockade such as colitis or rash is approximately 20% for nivolumab or pembrolizumab (anti-PD-1) treated patients and 28% in ipilimumab (anti-CTLA4)-treated patients. IrAE incidence increased to about 60% when pembrolizumab or nivolumab is combined with ipilimumab.^4 5 7^ Notably, the safety profile improves by reducing the dose of ipilimumab used in combination with nivolumab in metastatic renal cell carcinoma and melanoma.^8 9^ The biological mechanisms driving irAE in this setting are not fully known, but a common hypothesis is a combination of increased T-cell effector function driven by dual immune checkpoint blockade and depletion of regulatory T-cells (Tregs) driven by anti-CTLA4 antibody-dependent cytotoxicity (ADCC) mechanisms are key contributors.^10 11^ It has been difficult to study the mechanisms involved in checkpoint blockade-mediated irAE due to a lack of preclinical models that mirror these clinical adverse effects.

Preclinical studies in mouse tumor models have supported the molecular design of PD-1 and CTLA4 antibodies that are approved for clinical use. Specifically, binding activating FcγRs and eliciting FcγR-mediated effector functions such as ADCC is not desired for the anti-PD-1 antibody class to avoid depletion of the beneficial antitumor CD8+ effector T-cells that express PD-1. Antitumor responses driven by surrogate anti-mouse PD-1 antibodies require no Fc effector function and surrogate antimouse PD-1 antibodies that do engage activating FcγRs (ie, mouse IgG2a isotype) have no antitumor activity.^12^ Accordingly, the anti-PD-1 antibodies used in clinical practice use the human IgG4 isotype with reduced activating FcγR effector function.^13^

In contrast, mouse studies indicate that activating FcγR binding is required to achieve monotherapy antitumor efficacy with surrogate anti-mouse CTLA4 antibodies.^14 15^ Mouse antitumor immune responses are particularly sensitive to the presence of Tregs^16 17^ and anti-CTLA4 antibodies that bind activating FcγR (ie, mouse IgG2a isotype) effectively deplete tumor Tregs which express more CTLA4 on their cell surface compared with effector T-cells.^15 16^ It is unclear whether tumor Treg depletion occurs in humans following anti-CTLA4 antibody dosing and, if so, what contribution Treg depletion contributes to efficacy.^18–20^ Ipilimumab, approved for use in monotherapy and in combination with anti-PD-1, has the human IgG1 isotype with potent binding to activating FcγRs.

We examined the contribution of the Fc portion of surrogate anti-mouse CTLA4 antibodies in driving both antitumor efficacy as well as gut inflammation with the hypothesis that the antitumor requirement for anti-CTLA4 antibody’s Fc-effector function will be circumvented when combined with PD-1 blockade. A key impediment for assessing the influence of Fc-effector function on the induction of gut inflammation in syngeneic tumor models has been the lack of measurable inflammation and colitis using surrogate anti-mouse CTLA4 antibodies. Thus, we developed a novel model of enterocolitis mediated by anti-CTLA4 treatment in mice. We show that Fc-effector function is required for anti-CTLA4-driven intestinal inflammation and the inflammation is further exacerbated on combination treatment with anti-PD-1. Furthermore, immune checkpoint blockade-induced intestinal inflammation is driven by effector T-cells and macrophages but is independent of Treg depletion. Finally, CTLA4 antagonists that lack Fc effector function (Fc-mutant antibody or Fc-null single variable domain on a heavy chain (VHH) antibody) do not elicit intestinal inflammation yet can still drive a strong antitumor response when combined with anti-PD-1. Our results suggest CTLA4 blocking agents that enable greater efficacious dosing without increasing immune-related toxicity could be an effective therapy option to be used in combination with PD-1 blockade in the clinical setting.

## Results

### Anti-CTLA4 antibody Fc-effector function is required for gut inflammation induction

**Figure 1:**
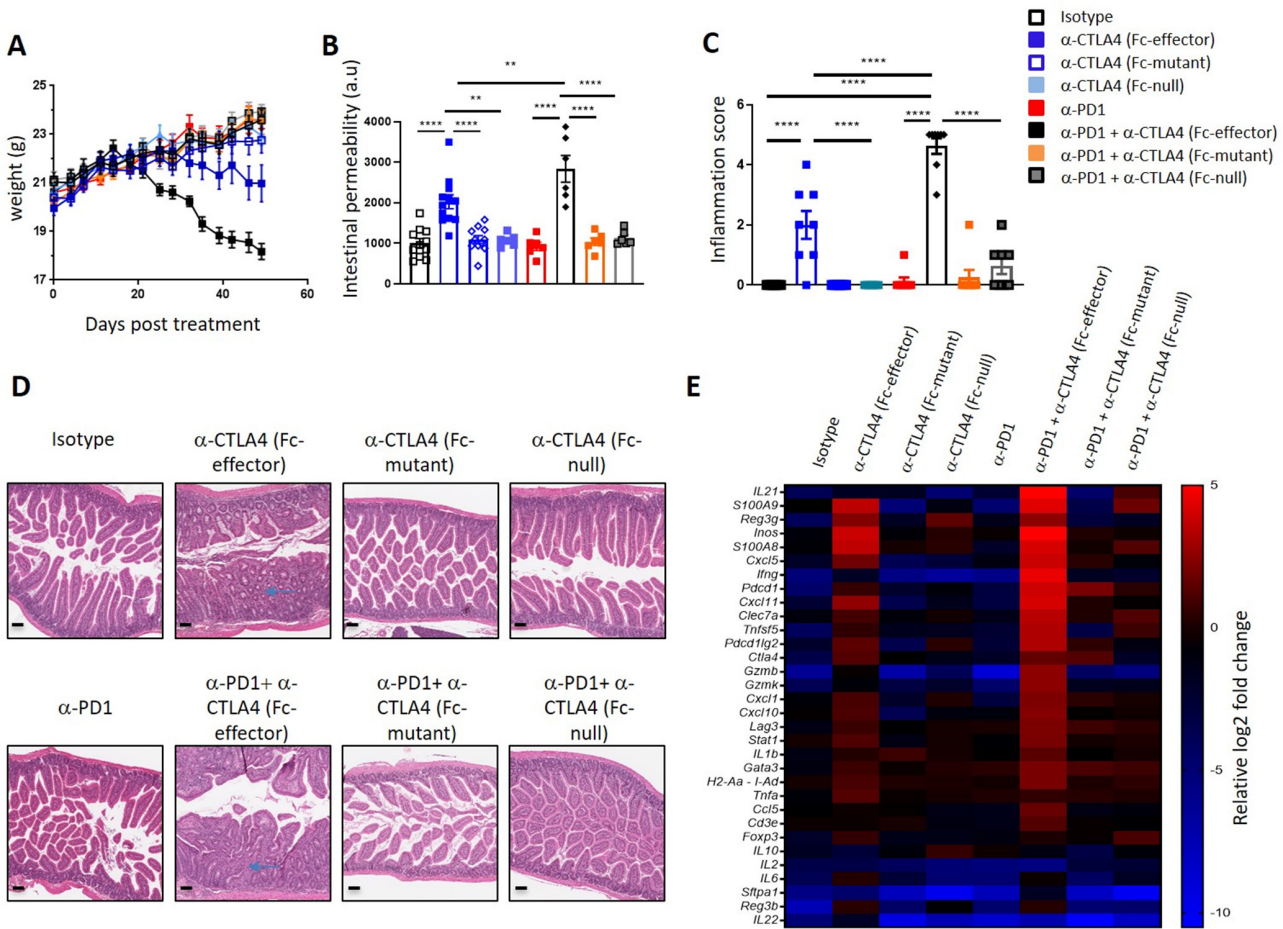
Anti-CTLA4-mediated intestinal inflammation is Fc-dependent and more severe in combination with PD-1 blockade. Balb/c mice were treated twice a week with antibodies as indicated for 50 days. (A) Mean body weights over the time. (B) Intestinal permeability assessed by fluorescence measurement of Fluorescein isothiocyanate (FITC)-dextran in the serum at day 50. a.u. Arbitrary units. (C) Intestinal inflammation score and (D) representative photomicrographs of H&E stained histological section of the small intestine at day 50 (n=8 mice/ group). Scale bars represent 100 µm. (E) RT-qPCR gene expression in small intestine at day 50 post treatment. Results are shown as mean log2 fold change relative to isotype control treated mice. Results are representative of two independent experiments (n=8 mice per group). ***P<0.001, ****p<0.0001 (one way analysis of variance test). Error bar ±SEM.

To test the requirement of FcγR function for anti-CTLA4-mediated irAE, we used three CTLA4 antagonists with different abilities to interact with FcγRs henceforth referred to Fc-effector (antibody clone 9D9 on a mouse IgG2a isotype with full ability to bind activating FcγRs), Fc-mutant (antibody clone 9D9 on a mouse IgG1 backbone with a D265A mutation that eliminates binding to FcγRs), and Fc-null (Fc-less bivalent anti-CTLA4 VHH coupled to an albumin-binding VHH for half-life extension). Anti-CTLA4 treatments exacerbate existing colitis in mouse models that employ irritants which induce intestinal epithelial damage such as dextran sodium sulfate, but no model to date has demonstrated that anti-CTLA4 treatment can drive intestinal inflammation de novo.^21 22^ Deletion of CTLA-4 during adulthood leads to autoimmune disease in mice^23^; therefore, we hypothesized that prolonged CTLA-4 neutralization might mimic irAE observed in clinic. Treatment of naïve Balb/c mice biweekly with Fc-effector anti-CTLA4 antibody for 7 weeks resulted in weight loss (figure 1A) associated with increased intestinal permeability (figure 1B) and severe enterocolitis (figure 1C–E). Histological inflammation progresses from the small intestine to the colon and was associated with immune cell infiltration in the lamina propria and mucus release into the lumen with mild diarrhea. No obvious immune cell infiltration was observed in kidney, liver, or lung after prolonged treatment with Fc-effector anti-CTLA4 (online supplemental figure 1A). Proinflammatory genes such as *Il1b*, *Tnfa*, *Ifng*, *Stat1*, *Il22* and *Inos* were upregulated in the colon from mice treated with Fc-effector anti-CTLA4 as early as 10 days post treatment (online supplemental figure 1B) before histological disease was observed (online supplemental figure 1C). Combination of Fc-effector anti-CTLA4 antibody with anti-PD-1 antibody significantly aggravated intestinal inflammation (figure 1A–E) as mice started to lose weight 20 days post-treatment initiation. Importantly, intestinal inflammation was not observed when mice were dosed with the Fc-mutant anti-CTLA4 antibody or Fc-null anti-CTLA4 VHH with anti-PD-1 demonstrating that FcγR function is required for Fc-effector anti-CTLA4-mediated intestinal inflammation (figure 1, online supplemental figure 1).

10.1136/jitc-2020-001584.supp1 Supplementary data

### T cells and macrophages are key drivers of anti-PD1/anti-CTLA4-mediated intestinal inflammation

**Figure 2:**
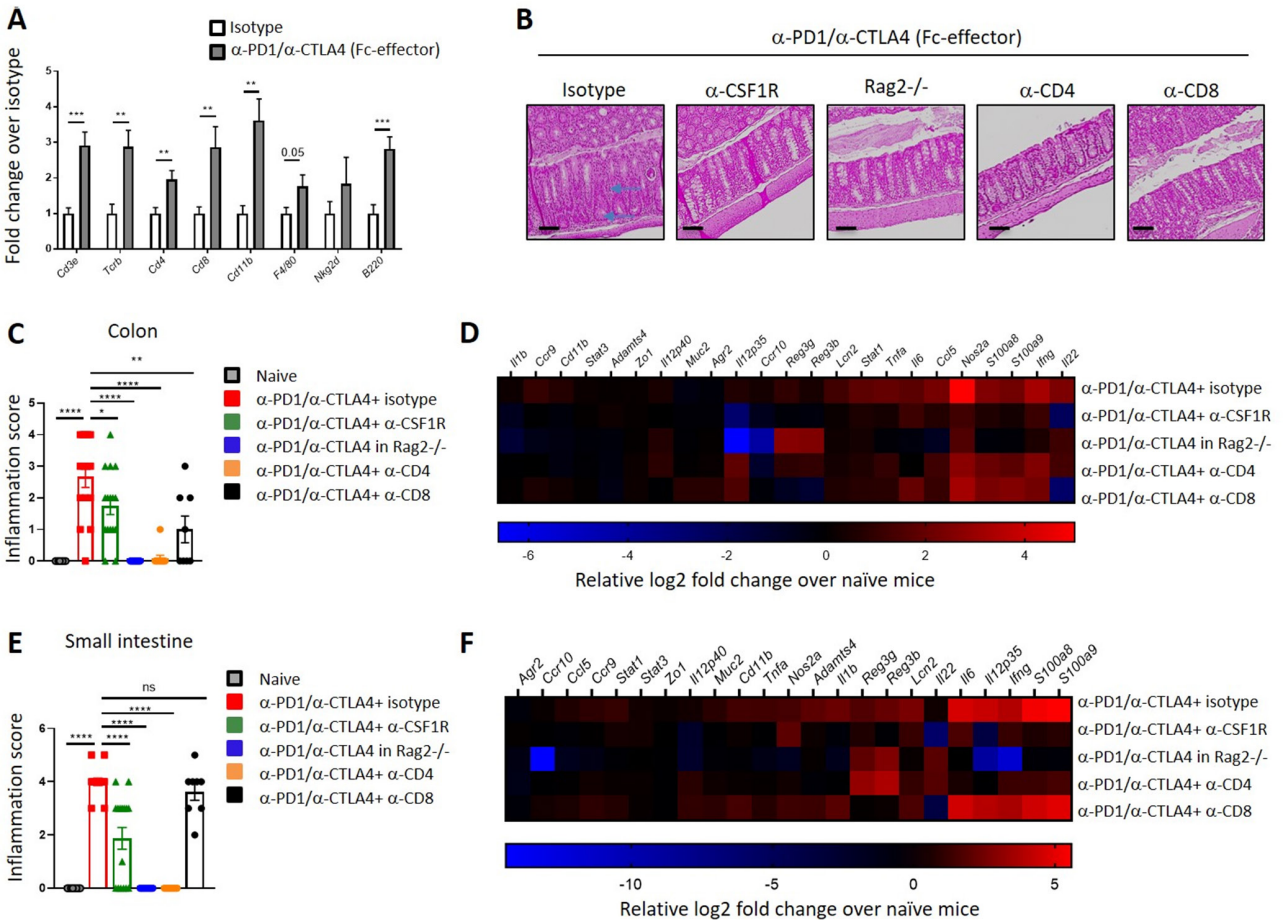
T cells and macrophages are key drivers of anti-PD1/anti-CTLA4-mediated intestinal inflammation. (A) Log fold change gene expression in colon comparing isotype control to anti-PD1/anti-CTLA4 (Fc-effector) treated mice at day 35 post treatment. (B–D) Balb/c mice were treated twice a week with antibodies as indicated for 34 days. (B) Representative photomicrographs of H&E stained histological section of the colon and (C) colon inflammation score. Arrows indicate immune cell infiltration and loss of goblet cells, indicative of inflammation. Scale bars represent 100 µm. (D) Fold change gene expression in colons comparing naïve mice to day 34 post-treatment groups. Log2 mean fold change values are represented. (E) Small intestine inflammation score. (F) Log2 fold change gene expression in small intestine comparing naïve mice to day 34 post-treatment groups. Log2 mean fold change values are represented. Results are representative of 2–3 independent experiments (n=8–16 mice per group). ns: not significant *p<0.05, **p<0.01. ***p<0.001, ****p<0.0001 (unpaired t-test for panel A and one-way analysis of variance test for panel C) and E). Error bar ±SEM.

Prior reports suggest that macrophages and T-cells play crucial roles during anti-CTLA4-mediated antitumor responses in mice.^24^ Gene expression profiling of colons from anti-PD-1 and Fc-effector anti-CTLA4 treated mice revealed an increased expression of genes associated with T-cells (*Cd3e, Tcrb, Cd4, Cd8*), macrophages (*Cd11b, F4/80*), NK cells (*Nkg2d*), and B cells (*B220*) indicating these cell types constitute the inflammatory infiltrate recruited to the colon (figure 2A). Predictably, combination anti-PD1/anti-CTLA4 treatment did not cause gut inflammation in RAG2 knockout mice which do not have B or T cells (figure 2B, C). We performed depletion studies to further parse the contribution of different immune cell types in mediating combination anti-PD-1/CTLA4 (Fc-effector) driven inflammation. Depletion of CD4 T-cells and macrophages reduced combination anti-PD-1/CTLA4 (Fc-effector)-mediated colitis (figure 2B–D) and inflammation of the small intestine (online supplemental figure 2). T-cell and macrophage depletion was associated with reduced expression of colitis-associated genes such as *Il22*, *Ifng*, *Stat1* and *Tnfa* (figure 2E). Depletion of CD8 T-cells reduced inflammation in the colon, but not the small intestine. (figure 2B–E and online supplemental figure 2). Overall, these data indicate T-cells and myeloid cells contribute to intestinal inflammation driven by immune-checkpoint blockade in this model.

### Fc-effector anti-CTLA4 antibody does not deplete colon lamina propria Tregs

Depletion of Tregs within the tumor microenvironment is one proposed mechanism for Fc-effector anti-CTLA4 antibody antitumor efficacy in murine syngeneic tumor models.^14 15 24^

**Figure 3:**
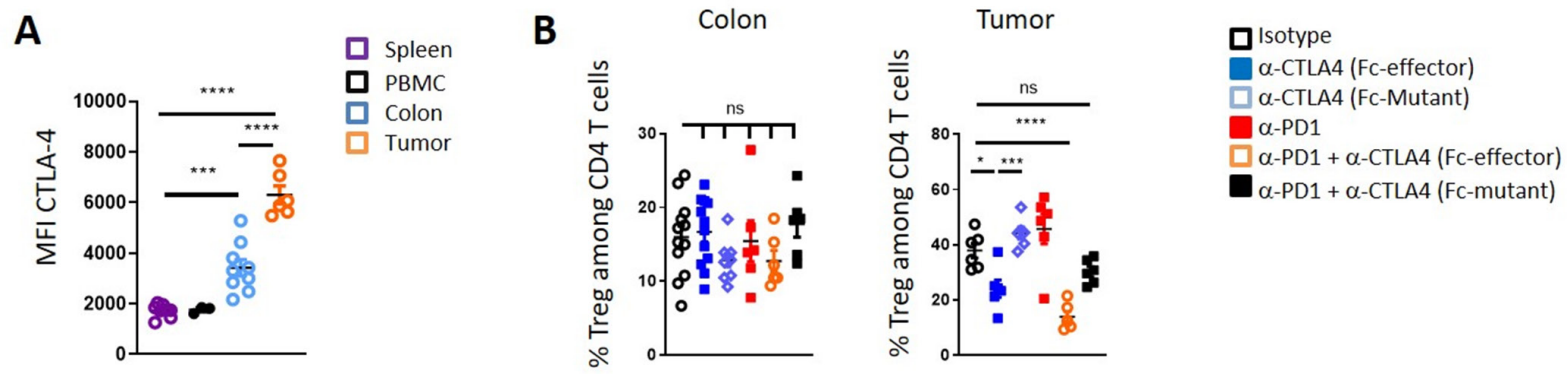
Fc-effector anti-CTLA4 antibody does not deplete colon lamina Tregs. (A) Mean fluorescence intensity (MFI) of intracellular CTLA4 in Foxp3^+^ Tregs from indicated organs and peripheral blood mononuclear cell (PBMC) from CT26 tumor-bearing mice when tumors were ~100 mm^3^. (B) Proportion of colon lamina propria and CT26 tumor-infiltrating Foxp3^+^ Treg 24 hours after treatment as indicated. Results are representative of 2–3 independent experiments (n=6–12 mice per group) ns: not significant, *p<0.05, ***p<0.001, ****p<0.0001 (one way analysis of variance test).

Differential expression of CTLA4 on various T cell populations impacts the capacity for Fc-effector anti-CTLA4 antibody to kill via receptor density-dependent ADCC. In agreement with previous reports,^14^ we observed differential CTLA4 expression by Tregs in spleen and tumors of CT26 tumor bearing mice, while Tregs from the colon lamina propria express an intermediate level of CTLA4 (figure 3A). Significant Treg depletion was limited to tumor infiltrating lymphocytes in mice treated with Fc-effector anti-CTLA4, which have the highest density of CTLA4 expression. Similar to ipilimumab-treated patients,^25^ no significant Treg depletion was detected in the colon lamina propria of Fc-effector anti-CTLA4 antibody treated mice (figure 3B) despite intermediate CTLA4 expression levels. Additionally, no significant changes in genes involved in Treg function such as *Foxp3*, *Tgfb1*, *Il10*, *Cd25* were observed in sorted colon lamina propria Tregs from Fc-effector anti-CTLA4-treated mice (online supplemental figure 3).

**Figure 4:**
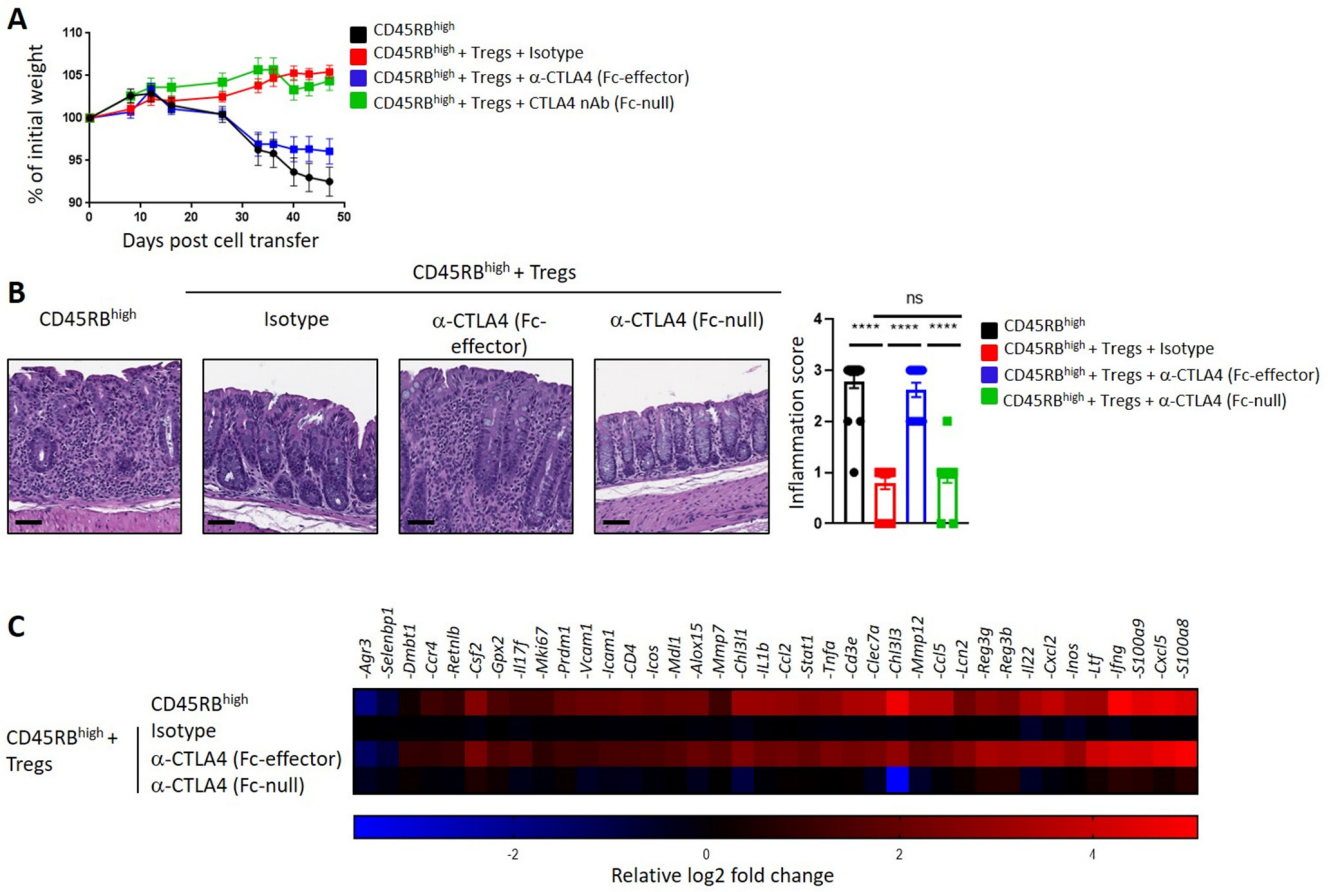
Fc-effector anti-CTLA4 impaired Treg-mediated suppression of colitis. Splenic CD45Rb^high^ naïve T cells and CD25 +Tregs were transferred into CB17-SCID (Severe combined immunodeficiency) recipient mice and treated with Fc-effector anti-CTLA4 antibody or Fc-null CTLA4 VHH antagonist as indicated. (A) Mouse weight over the time of the experiment. (B) Photomicrographs of H&E stained histological sections of the colon (left panel) and pathology score (right panel) at day 47 (n=14–18 mice per group). Scale bars represent 50 µm. (C) Fold change gene expression in whole colon comparing to day 47 post naïve T cell transfer (n=6 mice per group) treated groups. Data are representative of two independent experiments. ns: not significant ****p<0.0001 (One way analysis of variance test). Error bar ±SEM.

We next used a model of colitis where Tregs have a well-documented role in suppressing colonic inflammation in order to better understand the relative impact of anti-CTLA4 Fc effector function versus CTLA4 blockade on Treg activity. Naïve CD4 T cells (CD45Rb^high^ CD4 T-cells) were transferred with or without Tregs into CB17-SCID recipient mice. Mice developed colitis 6 weeks post transfer when naïve T cells were administered alone, while Treg co-transfer prevented colitis (figure 4A–C) in agreement with previous reports.^26^ Fc-effector, but not Fc-null, anti-CTLA4 treatment impairs Treg-mediated suppression of colitis (figure 4A–C). Altogether, these data suggest that functional blockade of CTLA4 does not exacerbate colitis per se; FcγR engagement by Fc-effector anti-CTLA4 is required to drive intestinal irAE.

### Anti-CTLA4 Fc-effector function is not required for antitumor efficacy in combination with anti-PD-1

**Figure 5:**
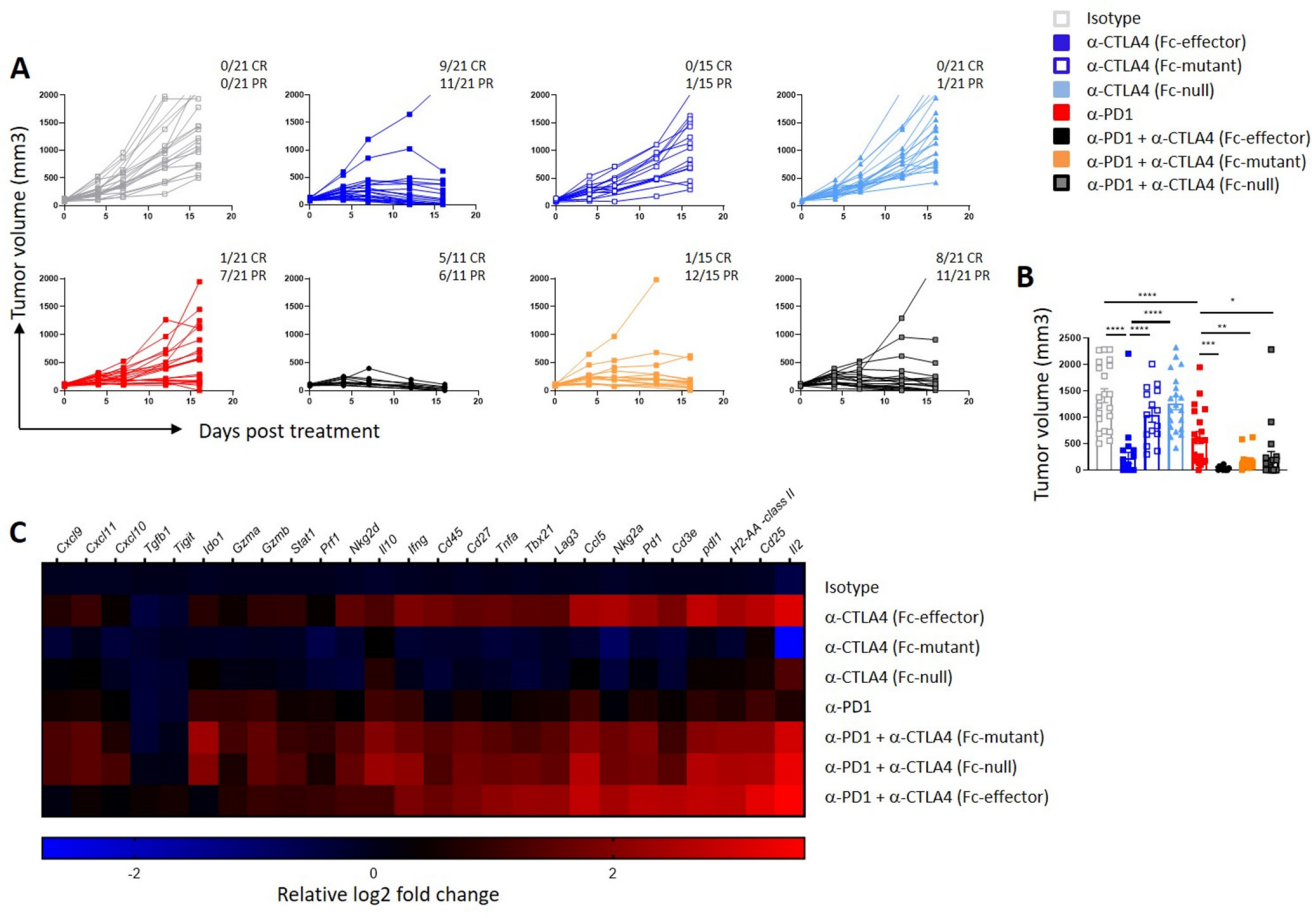
CTLA4 antagonists have a potent antitumoral efficacy only in combination with anti-PD-1. (A) CT26 tumor-bearing mice received the indicated antibody (at 20 mg/kg) or VHH (30 mg/kg) q4d×5 when tumors reached an average size of 100 mm^3^ (range 78–125 mm^3^). (B) Tumor volumes at day 16. The combined results from three independent experiments (n=11–21 mice per group) are shown. (C) Gene expression in whole tumor comparing isotype control treated mice to day 8 post-treatment groups Mean log2 fold change values re-represented. Results are representative of two independent experiments (n=5 mice per group). *P<0.05, **p<0.01, ***p<0.001, ****p<0.0001 (unpaired t-test). Error bar ±SEM.

Antitumor efficacy induced by the three CTLA4 antagonists with or without anti-PD-1 was assessed in the syngeneic CT26 colon carcinoma tumor model (figure 5) as well as in MB49 and MC38 tumor models (online supplemental figure 4). No antitumor activity was observed in monotherapy cohorts treated with either Fc-mutant or Fc-null CTLA4 antagonists (figure 5A, B). In contrast, strong monotherapy antitumor activity was observed in mice treated with Fc-effector anti-CTLA4 (figure 5A, B) consistent with prior reports.^15^ Treatment with monotherapy anti-PD-1 provided partial tumor growth inhibition. Anti-PD-1 combination with either Fc-mutant or Fc-null CTLA4 antagonists provided strong antitumor benefit despite of the absent Fc-function.

Gene expression profiling of tumors from mice treated with monotherapy Fc-effector anti-CTLA4 that depleted Tregs (figure 4B) showed strong upregulation of genes associated with effective immunotherapy including *Ifng*, IFN-response genes, chemokines, proinflammatory cytokines and major histocompatibility complex (MHC) (figure 5C). Monotherapy treatment with the two pure CTLA4 antagonists, Fc-null and Fc-mutant anti-CTLA4, and monotherapy anti-PD-1 demonstrated modest upregulation of these genes. In the anti-PD-1 combination setting, robust upregulation of tumor immune response genes was observed in all cohorts including mice treated with Fc-null or Fc-mutant pure CTLA4 antagonists (figure 5C). These data support the hypothesis that combining CTLA4 antagonism with PD-1 antagonism merges complementary mechanisms to provide strong combination antitumor benefit in a manner that does not require FcγR engagement which can be detrimental in other organs such as the gastrointestinal track.

## Discussion

irAE are a common side effect in patients undergoing immune checkpoint blockade for cancer, with colitis being the most serious and sometimes fatal irAE associated with anti-CTLA4 treatment.^27^ Surprisingly, there is a paucity of translational models that mimic anti-CTLA4-related clinical adverse events. Surrogate anti-CTLA4 treatment enhances Dextran sulfate sodium (DSS)-induced colitis—a model of ulcerative colitis without perforation—in C57BL6 mice but the inflammation observed does not mimic enterocolitis described in patients.^21 22^ We show that long-term treatment with an Fc-effector anti-CTLA4 antibody twice weekly in Balb/c mice induces enterocolitis with similar T-cell gene signature to what has been observed in the clinic with ipilimumab and tremelimumab.^28–30^ Unlike clinical observations, no other irAE were observed in Fc-effector anti-CTLA4 antibody. As previously described, it is important to note that C57BL/6 mice are less prone to developing Fc-effector anti-CTLA4-mediated colitis compared with Balb/c mice (personal observations and 31). While colitis development following anti-PD-1 treatment is less common (2% of patients) compared with anti-CTLA4 (11%) in clinical studies, the combination of the two immunotherapies increases both the incidence (13%) and severity of colitis in patients with melanoma.^4^ Notably in our model, monotherapy anti-PD-1 treatment does not elicit gut inflammation but exacerbates the colitogenic effects of Fc-effector anti-CTLA4. Interestingly, monotherapy Fc-effector anti-CTLA4-induced inflammation in mice is restricted to the gut; however, immune cell infiltration was detected in the lung and liver following combination with anti-PD-1 mimicking what has been observed in the clinical setting.^4^

Fc effector function is required for antitumor efficacy of anti-CTLA4 antibodies in mouse tumor models and antibody-mediated depletion of tumorous, but not peripheral, Tregs has been shown to be a critical factor.^14 15^ We have shown the requirement for anti-CTLA4 Fc effector function is eliminated if Fc effector-less CTLA4 antagonists are combined with anti-PD-1 in mouse tumor models. These data suggest that enhanced antitumor efficacy can be achieved solely via activation of T cells through checkpoint antagonism and does not require antibody-mediated Treg depletion or other Fc-mediated functions. Furthermore, we demonstrate that elimination of FcγR engagement minimizes gut inflammation elicited during prolonged treatment with CTLA4 antagonists even in the context of PD-1 blockade, suggesting the potential for extending combination therapy benefits by reducing irAE incidence or delaying onset. Thus, in contrast to sole CTLA4 antagonism needed for combination antitumor activity Fc-effector function is required to induce immune-mediated colitis whereas CTLA4 antagonism alone is not sufficient. CD4 and CD8 T cells play a differential role in colitis and the antitumor response. While CD4 T cells are the main driver of colitis in our model and in patients who developed colitis after receiving anti-CTLA-4 treatment (figure 2 and^32^), CD8 T cells are required for antitumor efficacy in the three syngeneic models that we studied.^33–35^ Unlike CD8 T cells from colon lamina propria, tumor-infiltrating CD8 T cells express high level of CTLA-4 and PD-1^36^; therefore, we hypothesized that the differential expression or requirement of CTLA-4 in other immune cell subsets such as CD8 T cells or conventional CD4 T cells can explain this observation.

While Tregs protect against T cell-mediated gut inflammation in mouse models and intestinal Tregs express higher levels of CTLA4 than peripheral Tregs, we found no evidence suggesting Fc-effector anti-CTLA4 drives intestinal Treg depletion. This is in agreement with similar observations in colon biopsies from ipilimumab-treated patients where Treg depletion was not apparent.^25 29 32 37^ and with studies in adult mice which show CTLA4 expression on Tregs is neither necessary for their suppressive function nor critical for preventing systemic inflammation.^38^ Both colitis models taken together suggest that the inflammatory mechanism of Fc-effector anti-CTLA4 in the context of FcγR engagement is driven more by effector T-cells than Tregs. Depletion studies show that both T-cells and macrophages are cellular mediators of intestinal inflammation in our model. Waight *et al* demonstrated that FcγRIIIA (in human) and FcγRIV (in mouse) engagement is crucial for an optimal effector T-cell response on anti-CTLA4 treatment.^24^ Although our data suggest that FcγR coengagement on macrophages may also contribute to intestinal irAE, further studies are required to better understand FcγR coengagement in irAE.

Notably, ipilimumab is an IgG1 antibody (known effector function) while tremilimumab is an IgG2 antibody (associated with weak to no effector function) but these two antibodies have similar binding properties to CTLA4^39^ and toxicity profiles.^3 40^ It was previously demonstrated that intestinal myeloid cells express a high level of activating FgRII at their surface^41 42^ and anti-CTLA-4 antibodies on both IgG1 or IgG2 backbones can induce ADCC.^43^ We therefore speculate that both tremilimumab and ipilimumab can equally engage FcgR-expressing myeloid cells leading to similar adverse events including enterocolitis.

In summary, we describe a model of immune checkpoint blockade-driven gut inflammation that is dependent on FcγR engagement of anti-CTLA4 antibody. By avoiding FcγR receptor engagement, we minimize gut inflammation but maintain potent combination antitumor efficacy with anti-PD-1. This model will be useful in further determining mechanisms that drive immunotherapy-associated colitis as well as designing mitigation strategies to prevent immune-related colitis from developing. One strategy is to avoid FcγR engagement by CTLA4 antagonists.

## Materials and methods

### Mice

Wild-type C57BL/6J and Foxp3-GDL^44^ mice were obtained from Jackson laboratories. Wild-type Balb/cAnNTac, C.129S6(B6)-*Rag2^tm1Fwa^* N12 mice and C.B-17 scid mice were obtained from Taconic. Mice maintained under specific pathogen-free conditions and kept in microisolators with filtered air at the research laboratories of Merck & Co., Palo Alto and South San Francisco, California, USA (MRL) animal facilities.

### Antibodies

Clone 9D9^14^ was engineered onto mouse IgG2a isotype to produce Fc-effector anti-CTLA4 antibody and engineered onto mouse IgG1 isotype with D265A substitution to eliminate Fc receptor binding to produce Fc-mutant anti-CTLA4.^45^ Clone DX400 engineered onto mouse IgG1 isotype with D265A mutation to produce anti-PD-1 was published previously.^46^

The anti-mouse CTLA4 VHH (referred to as Fc null anti-CTLA4) was identified by Ablynx and consists of three camelid VHH domains covalently linked in tandem by Gly/Ser linkers. Two of the VHH subunits are specific for mouse CTLA4 and the third binds to mouse albumin to serve as a half-life extension. The CTLA4 VHH antagonist was selected from a VHH library derived from llamas immunized with mouse CTLA4 DNA after screening for binding to mouse CTLA4-transfected CHO cells and competition of mouse CTLA4 binding to mouse CD80-Fc and CD86-Fc. The CTLA4 VHH antagonist had comparable blocking activity on CD80 and CD86 to the reference anti-CTLA4 antibody 9D9. Addition of the CTLA4 VHH antagonist to a mixed-lymphocyte reaction assay increased T cell proliferation, IFNγ and IL-2 secretion in vitro.

Antibodies used for in vivo cell depletion studies were from BioXcell: anti-CD4 (Clone GK1.5), anti-CD8 (Clone YTS 169.4), anti-CSF1R (Clone AFS98), Rat IgG2b isotype (Clone LTF-2), Mouse IgG2a isotype (Clone C1.18.4). Antibodies used for flow cytometry were from BD Biosciences, Biolegend, or eBioscience, and include CD45 (30-F11), CD8a (53-6.7), CTLA4 (UC10-4B9), TCRb (H57-597), CD4 (RM4-5 or GK1.5), CD25 (PC61), CD45RB (16A), F4/80 (T45-2342) and Foxp3 (FJK-16s).

### In vivo studies

For syngeneic tumor experiments, 8–12 week-old Balb/c mice were subcutaneously injected with either 10^6^ CT26, MC38 cells or 5.10^5^ MB49 cells in 100 μL on the right flank. Tumor diameter was measured using electronic calipers and tumor volume calculated using the equation 0.5 × length × width^2^, where the length was the longer dimension. Mice were randomized to treatment groups when tumors reached ~100 mm^3^. Mice were treated twice a week intraperitoneally (i.p.) with antibodies at 10 mg/kg and VHH at 30 mg/kg.

For antibody driven colitis studies, naïve 8–12 week-old Balb/c mice were treated twice a week i.p. for up to 8 weeks with anti-CTLA4 antibodies at 20 mg/kg, anti-CTLA4 VHH or control VHH at 30 mg/kg, and anti-PD-1 at 10 mg/kg. For immune cell depletion, mice were dosed i.p. with 500 μg at day 0, then twice weekly with 200 μg of the indicated antibodies.

For T cell-driven colitis studies, mouse CD4 T-cells were isolated from Balb/c spleens using magnetic bead separation (STEM CELL Technologies). TCRb^+^ CD4^+^ CD25^−^ CD45RB^high^ T cells (CD45RB^high^ T cells) and TCRb^+^ CD4^+^ CD25^+^ CD45RB^low^ (Tregs) were sorted with FACS Aria (BD). 3×10^5^ CD45RB^high^ T-cells and 1×10^5^ Tregs were injected intravenously. Mice were dosed i.p. twice weekly with 20 mg/kg of anti-CTLA4 antibody or 30 mg/kg of CTLA4 VHH. Mice were monitored and weighed for 7 weeks post injection.

For intestinal permeability studies, mice were gavaged with 1 mg of FITC-Dextran (4 kDa, Sigma-Aldrich) in 100 μl of sterile PBS and tail bleed 4 hours later. Serum FITC concentrations were measured using fluorescence Spectramax plate reader (Molecular Devices).

### Colon lamina propria and tumor cell isolation

Colon lamina propria cells were isolated by first removing epithelial cells through the incubation of 0.5 cm gut tissue pieces in Hank’s buffered salt solution containing 5 mM EDTA and 10 mM HEPES (4-(2-hydroxyethyl)-1-piperazineethanesulfonic acid) for 20 min at 37 °C and then repeating this incubation one additional time. The remaining tissue was cut into small fragments and then digested with HBSS 1× medium containing 0.250 mg/mL Liberase (Roche), 30 U/mL DNaseI (Sigma-Aldrich) and 50 U U/mL Dispase (Corning) at the same conditions. The resulting cell suspension was layered on to a 40%/80% Percoll gradient and centrifuged for 10 min at 600 *g*; Lamine Propria (LP) cells were recovered at the interface.

Subcutaneous tumors were excised, transfer in 5 mL of PBS and mechanically dissociated using gentleMACS dissociator (Miltenyi).

### Histology

Colon, liver, lung, and kidney were fixed in 10% neutral buffered formalin overnight, transferred to 70% ethanol, processed routinely, embedded in paraffin, sectioned at 4–5 µm, then stained with H&E. Colons were scored for severity of disease by a pathologist in a blinded fashion. The scoring of inflammation included severity of inflammatory cell infiltration, loss of glands, erosion, dilatation of glandular lumina, presence of crypt abscesses, and degenerated epithelial cells. Inflammatory cell infiltrate when present was characterized predominantly by mononuclear cells with a lower proportion of granulocytes. Inflammation was scored on a scale of 0–5, 0=negative; 1=minimal, 2=mild; 3=moderate; 4 severe, 5=extensive.

### Flow cytometry

Cells were resuspended in Phosphate-buffered saline (PBS) and stained on ice for 30 min in the dark with a fixable viability stain (BD Bioscience). Then, cells were resuspended into the stain buffer (bovine serum albumin, BSA) (BD bioscience) and stained on ice for 30 min with various combinations of directly fluorochrome-conjugated antibodies. For intracellular antigens, surface stained cells were fixed and permeabilized with Foxp3 staining buffer set (eBiosciences) for 30 min on ice and then stained with specific antibodies (refer to section antibodies). For all samples, acquisition was performed on LSR II flow cytometer (BD). Data were analyzed using FlowJo software (Tree Star).

### Total RNA isolation from tissues and cells and subsequent RT-QPCR gene expression analysis using the Fluidigm Biomark platform

For real-time PCR analysis, total RNA was isolated by either of two methods. Organs were homogenized in RNA STAT-60 (Tel-Test Inc.) with a polytron homogenizer and then RNA extraction was performed with the MagMAX-96 for Microarrays Kit (Thermo Fisher Scientific) per manufacturer’s instructions. For cellular samples, RNA was isolated using the ARCTURUS PicoPure RNA Isolation Kit per manufacturer’s instructions (Thermo Fisher Scientific).

DNase-treated total RNA was reverse-transcribed using QuantiTect Reverse Transcription (Qiagen) per manufacturer’s instructions. Primers were obtained commercially from Thermo Fisher Scientific. Gene specific pre-amplification was done on at least 2 ng cDNA per Fluidigm Biomark manufacturer’s instructions (Fluidigm). Real-time quantitative PCR was then done on the Fluidigm Biomark using two unlabeled primers at 900 nM each and 250 nM of FAM-labeled probe (Thermo Fisher Scientific) with Taqman Universal PCR Master Mix containing UNG. Samples and primers were run on either a 48.48 array or 96.96 array per manufacturer’s instructions (Fluidigm). Ubiquitin b levels were measured in a separate reaction and used to normalize the data by the ΔCt method. (Using the mean cycle threshold value for ubiquitin b and the gene of interest for each sample, the equation 1.8^(Ct ubiquitin b minus Ct gene of interest)^ × 10^4^ was used to obtain the normalized values.). Primer references sequences are available on demand.

### Statistics

One-way analysis of variance and unpaired t-test were used to calculate statistical significance in the rest of this study. Ns, not significant, *p<0.05, **p<0.01, ***p<0.001, ****p<0.0001. Statistics were performed using GraphPad Prism 7 software.
