# Supplementary material for: Antitumor efficacy of combined CTLA4/PD-1 blockade without intestinal inflammation is achieved by elimination of FcγR interactions
Source: J Immunother Cancer. 2020 Oct 30;8(2):e001584. doi: 10.1136/jitc-2020-001584 (PMC7604872; doi:10.1136/jitc-2020-001584)
Supplement: Supplementary data [file jitc-2020-001584supp001.pdf]

SUPPLEMENTARY MATERIALS

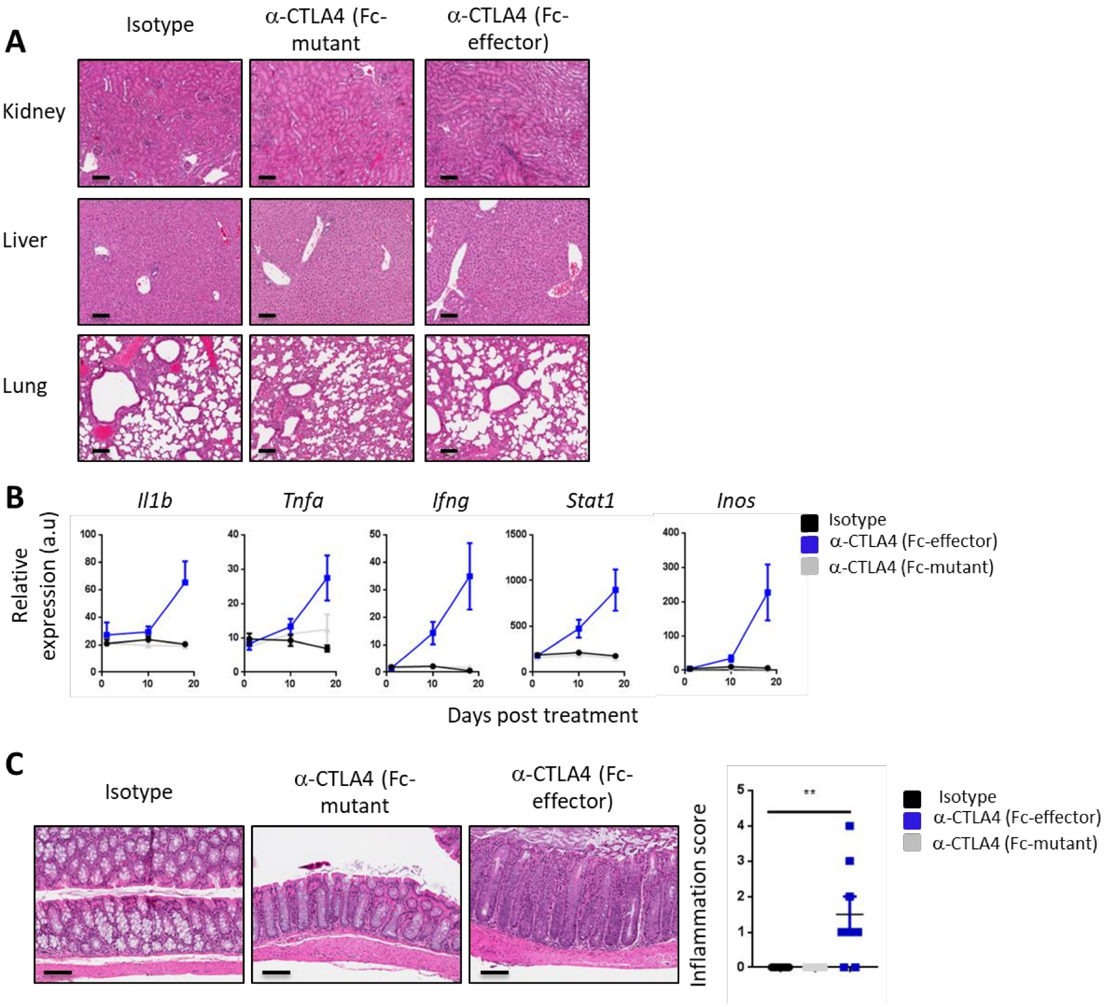

Supplementary Figure S1- Anti-CTLA4 blockade mediated intestinal inflammation is Fc-dependent
(**A**) Photomicrographs of H&E stained histological section of the kidney (top panel), liver (middle panel) and lung (bottom panel) (representative of 4-8 mice per group) at day 55 post treatment. (**B**) RT-qPCR gene expression of inflammatory genes from proximal colons at day 1, 10 and 18 following Fc-effector anti-mouse CTLA4 antibody CTLA4 antagonist administration (n=8-10 mice/ group). a.u: arbitrary units. (**C**) Balb/c mice were treated twice a week with antibodies as indicated for 55 days. Representative photomicrographs of H&E stained histological sections of colon and pathology score of treated mice at endpoint. Scale bars represent 100μm. Error bar +/- SEM. **P<0.01 (Unpaired t-test).

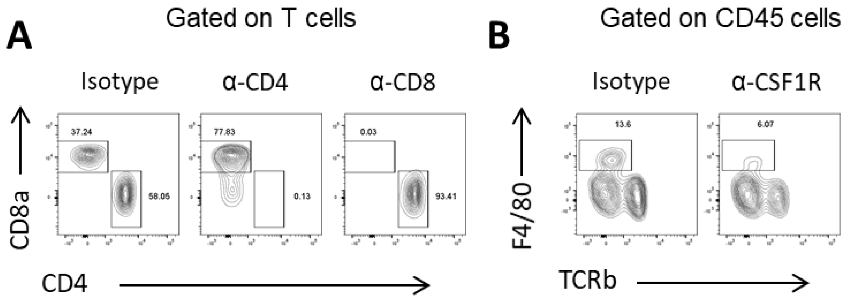

Supplementary Figure S2- T cells and Macrophages are key drivers of anti-PD1/anti-CTLA4-mediated intestinal inflammation

Balb/c mice were treated twice a week with antibodies as indicated for 34 days. (**A**) CD4, CD8 T cell and (**B**) macrophage depletion was measured by flow cytometry from the spleens of treated mice as indicated. Results are representative of 2 independent experiments (n=3 mice per group).

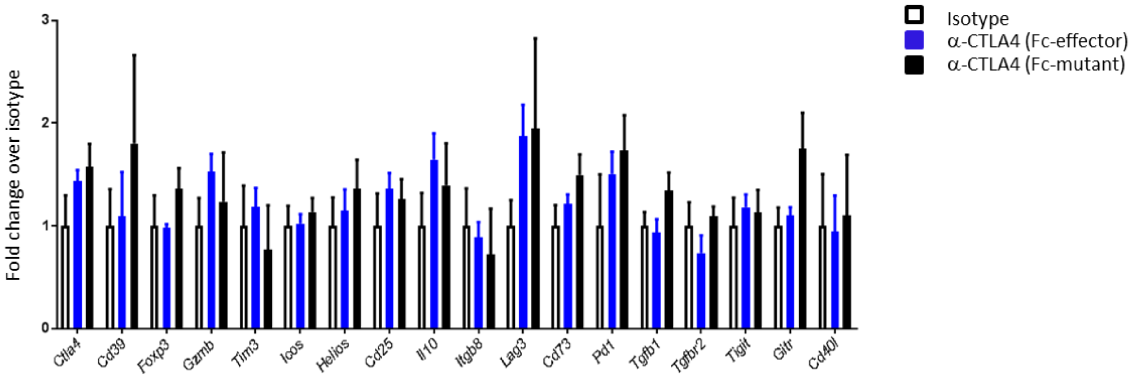

Supplementary Figure S3- CTLA-4 blockade does not alter genes associated with Treg function.

Fold change gene expression profile from flow cytometry sorted colon Foxp3-GFP Tregs mice 24 hours post treatment as indicated. Mean fold change comparing isotype treatment to 24 hours post treatment groups are represented from 2 independent experiments (n=4 mice per group). Error bar ± SEM

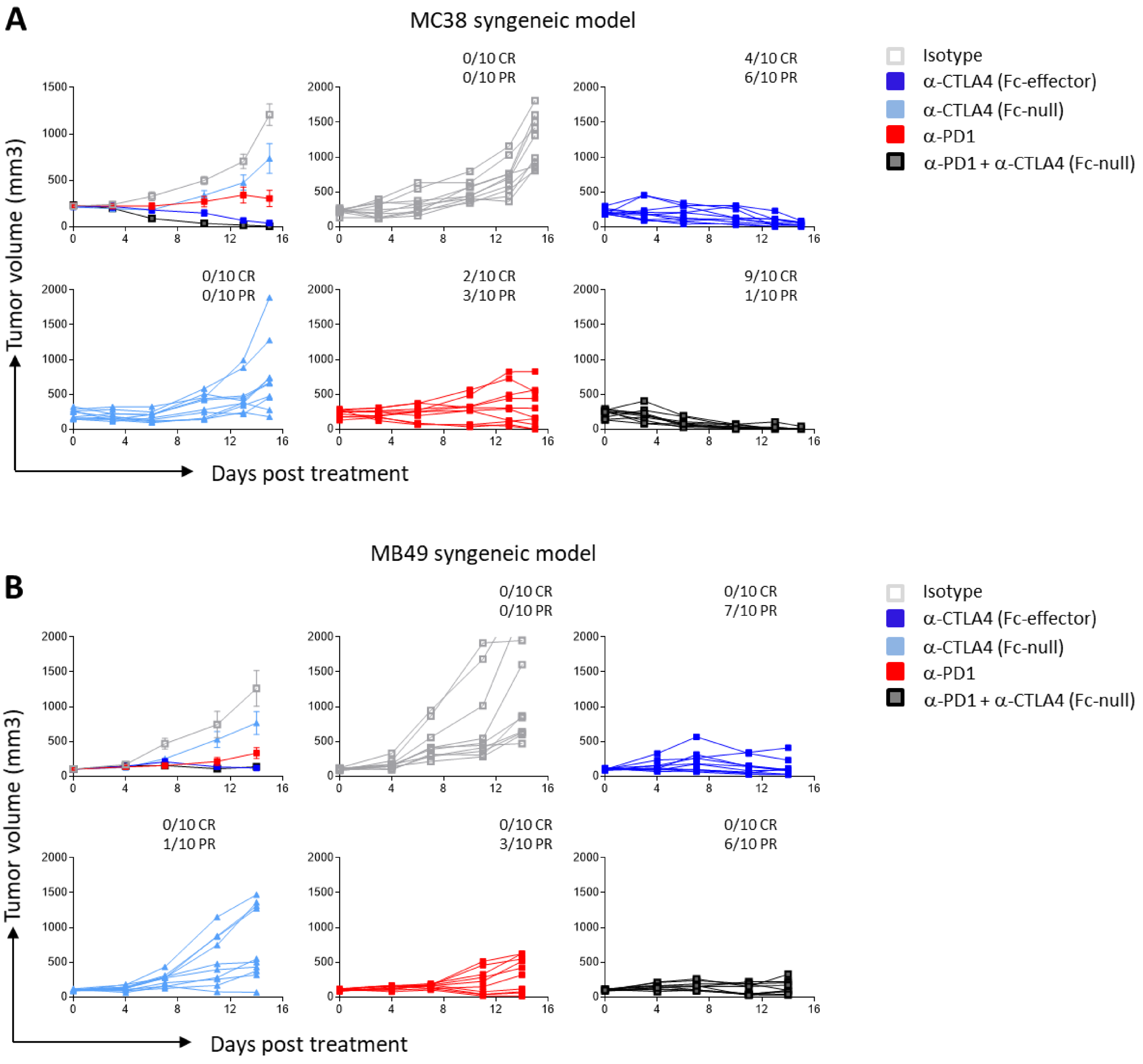

Supplementary Figure S4- CTLA4 antagonists have a potent anti-tumoral efficacy only in combination with anti-PD-1 in MC38 and MB49 syngeneic models.

(**A**) MC38 or (**B**) MB49 tumor-bearing mice received the indicated antibodies (at 20 mg/kg) or VHH (30 mg/kg) q4dx3 when tumors reached an average size of 100mm^3. Results are representative of 1 experiment (n=10 mice per group). Error bar ± SEM
